# Variation in the affinity of three representative avian adenoviruses for the cellular coxsackievirus and adenovirus receptor

**DOI:** 10.1186/s13567-024-01277-y

**Published:** 2024-02-19

**Authors:** Yapeng Song, Mingyue Tao, Lin Liu, Yang Wang, Zhenchao Zhao, Zongmei Huang, Wenming Gao, Qiang Wei, Xinsheng Li

**Affiliations:** 1 Department of Microbiology and Immunology, College of Veterinary Medicine, Henan Agricultural University, Henan, China; 2 Henan Provincial Key Laboratory of Animal Immunology, Henan Academy of Agricultural Sciences, Zhengzhou, 450002 China

**Keywords:** Avian adenovirus, fibre, crystal structure, coxsackievirus–adenovirus receptor, affinity variation

## Abstract

According to previous studies, three representative avian adenoviral strains utilize coxsackievirus–adenovirus receptor (CAR) as a receptor and seem to exhibit diverse binding affinities and modes. Thus, further revealing the exact molecular mechanism underlying the interaction between different FAdVs and the attachment receptor CAR is necessary. In this study, we successfully solved the crystal structure of the FAdV-4 fiber1 knob at 1.6 Å resolution. The interaction between the fibre knob and different domains of CAR was verified by confocal microscopy, coimmunoprecipitation and surface plasmon resonance (SPR) analysis. The fibre knobs of the three representative fowl adenoviruses specifically recognized CAR domain 1 (D1), but the recognition of CAR domain 2 (D2) by chicken embryo lethal orphan (CELO) strains was weak. These results provide insights into the differences in adenovirus‒host cell interactions and have important implications for the exploration of viral invasion mechanisms.

## Introduction, methods and results

Fowl adenovirus (FAdV) belongs to the family *Adenoviride* within the genus *Aviadenovirus*. FAdVs are nonenveloped icosahedral particles containing a linear double-stranded DNA genome [1]. Along with the hexon and penton base, the trimeric fibre protein is a major capsid protein, and all of these proteins are known to mediate binding to host cells. FAdVs are equipped with highly diverse capsid fibres that interact with receptors via their knob domains. FAdV-4 has almost equal length fiber1 and fiber2 proteins, which consist of 431 amino acids (aa) and 479 aa, respectively. In contrast, fiber1 of CELO has 710 aa, whereas fiber2 has 410 aa [2]. Unlike FAdV-4 and CELO, egg drop syndrome virus (EDSV) carries a single type of fibre protein. Previous studies have shown that the fibre knobs of CELO, EDSV and FAdV-4 play important roles in binding to the coxsackievirus-adenovirus receptor (CAR) [3–5].

CAR is a transmembrane protein that mediates cell–cell adhesion and contains two glycosylated extracellular Ig-like domains, D1 and D2, followed by a transmembrane domain [6] and an intracellular domain (ICD) [7]. CAR is a high-affinity primary receptor and coreceptor for human adenovirus (HAdV) groups A and C–F, as well as canine and gorilla adenovirus [8–10]. The D1 domain has been shown to be necessary and sufficient for high-affinity human adenovirus fibre knob binding [11]. Previous studies have demonstrated that the CELO virus, FAdV-4 and EDSV enter host cells by binding to CAR during infection, and the binding region and affinity between FAdVs and CAR are significantly different from those of HAdVs [2–4]. For instance, FAdV-4 utilizes fiber1 to bind to the CAR-D2 domain, which is different from the mechanism of other adenoviruses in which fibres bind to the CAR-D1 domain [2]. Therefore, further analysing the interaction between FAdV fibre knobs and the attachment receptor CAR in vitro is necessary.

Studies of receptor–virus interactions using structural biology techniques can significantly advance the understanding of FAdV attachment and entry. In this study, we report the structure of the FAdV-4 fiber1 knob at 1.60 Å resolution; this knob showed a similar structural conformation to the fiber1 knob of CELO and EDSV. Furthermore, the binding properties of the three representative FAdVs to CAR were assessed through a series of experiments. The results presented here provide insights into the plasticity of adenovirus‒host cell interactions.

## Expression and purification of the FAdV-4 fibre-knob protein

**Figure 1:**
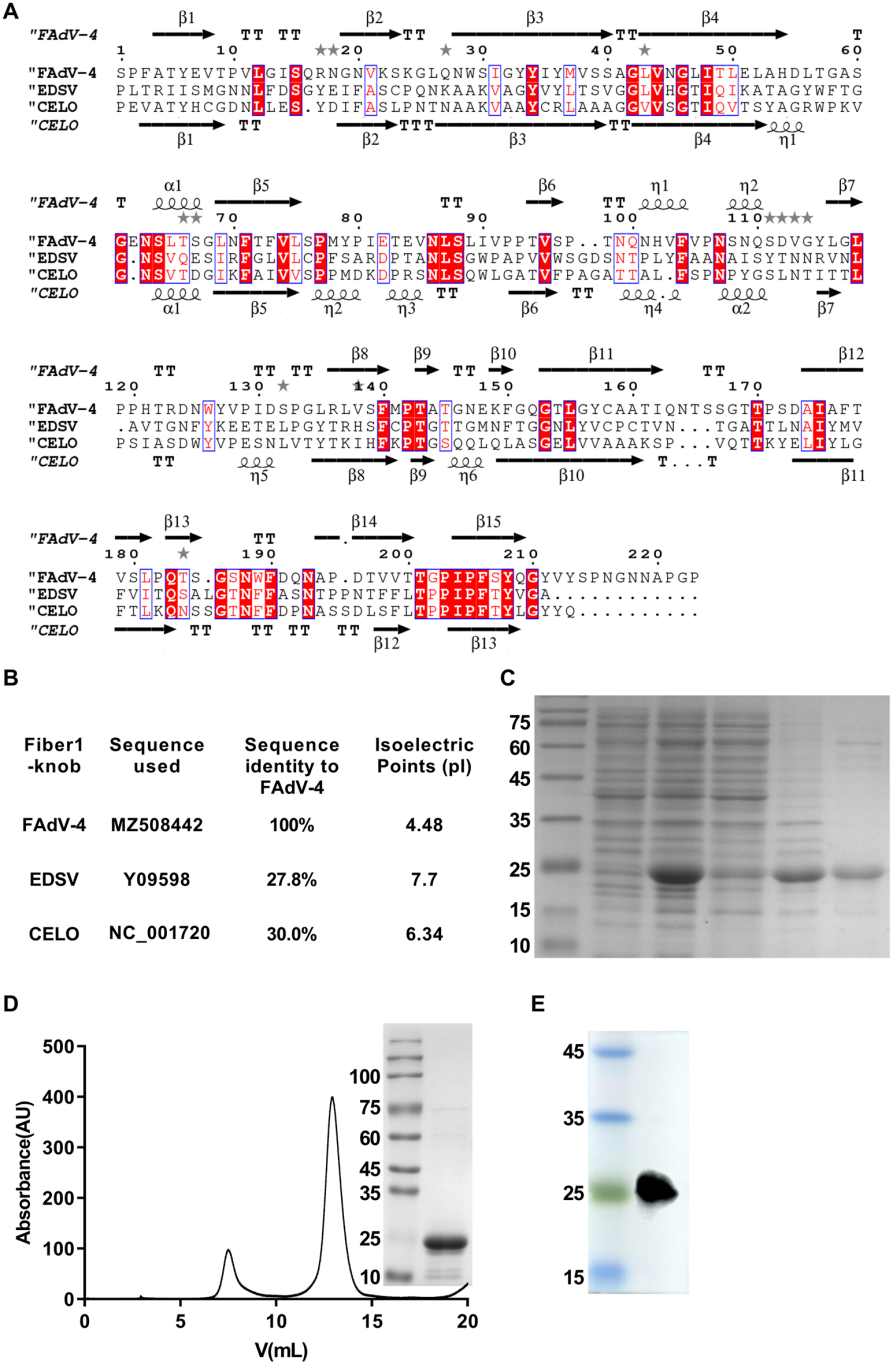
**Sequence alignment and expression of the F-F1K recombinant protein in the** ***E. coli*** **system.** **A** The secondary structures were determined via protein sequence analysis. The FAdV-4 strain sequence is shown at the top. **B** The homology and isoelectric points of the amino acids were determined. The purity of the F-F1K recombinant protein was analysed by SDS-PAGE (**C**) and Western blotting (**D**). **E** F-F1K protein was purified to high purity by a gel filtration column (Superdex 200 10/300 GL). SDS-PAGE analysis of the elution fraction corresponding to the peak (inset) was performed.

To investigate conservation of the fibre protein in terms of amino acid sequence and structure, we analysed the homology of the fibre knobs of three representative avian adenoviruses (Figure 1A). The predicted isoelectric points of the fibre knob proteins of the viruses were all lower than 7. This may indicate that the charge of the fibre knobs may play a role in virus‒cell surface interactions [12]. Notably, the three FAdVs were also highly similar in terms of their secondary structure.

To determine whether the FAdV-4 Fiber1 knob protein could be expressed in *E. coli* BL21 (DE3) cells, we analysed the protein expression of fiber1 in the collected cell lysates by SDS-PAGE and Western blotting (Figure 1C, D). The expression and purification of the fibre knobs were performed as described previously [3]. Western blot analysis revealed that the recombinant protein was found in chicken polyclonal serum against FAdV-4 (Figure 1E).

### Overview of the structure of the knob domain of FAdV-4 fiber1

**Figure 2:**
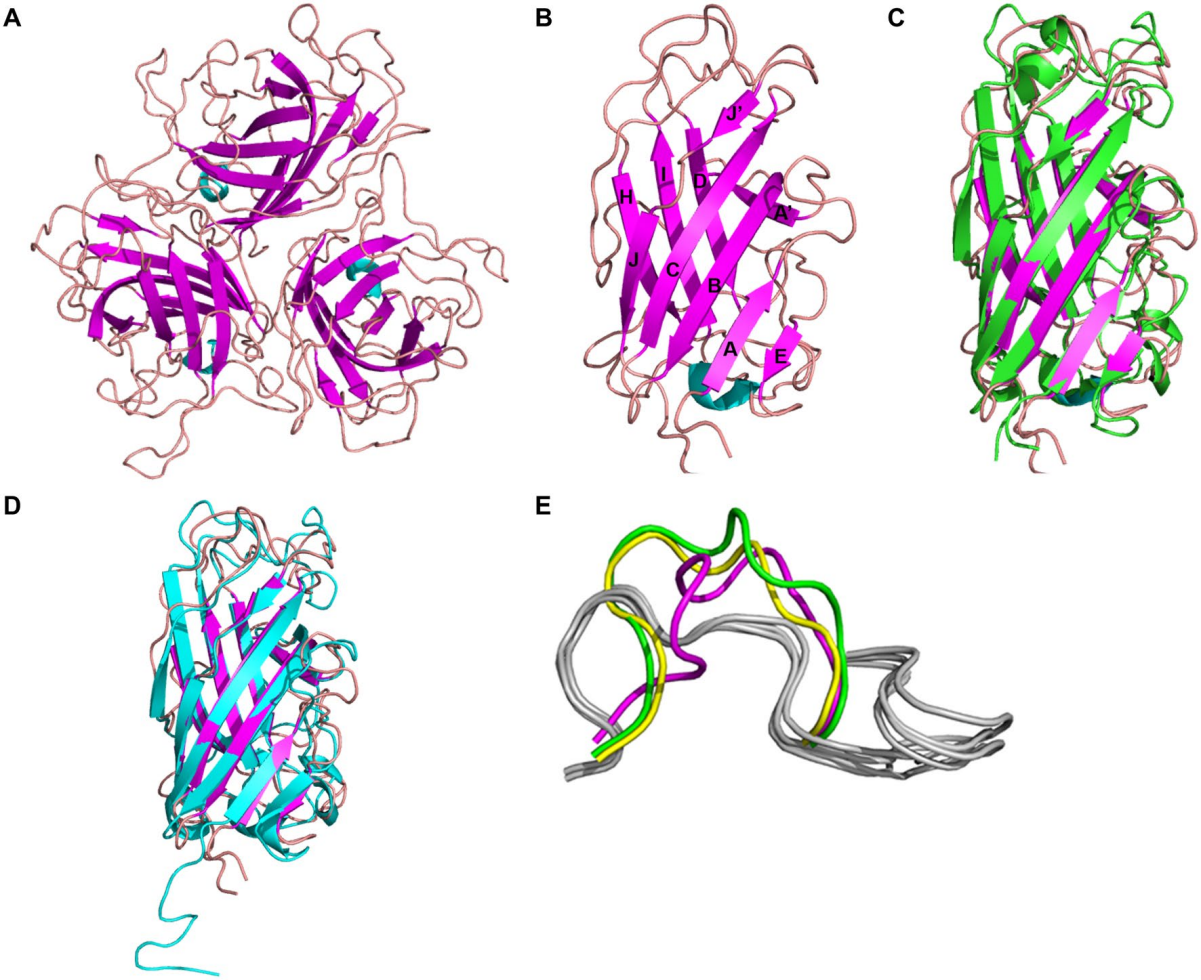
**Structure of the FAdV-4 fiber1 knob and comparison with other adenovirus fibre knob proteins.** **A** The crystal structure of the trimeric FAdV-4 fiber1 knob and **B** the monomeric FAdV-4 fiber1 knob are shown. **C** The FAdV-4 fiber1 knob was compared with the CELO fiber1 knob (PDB code 2IUN). The backbone of the FAdV-4 fiber1 knob is coloured magenta (**B**), while the backbone of the CELO fiber1 knob is coloured green. **D** The FAdV-4 fiber1 knob was compared with the EDSV fibre knob (PDB code 6ITX). The backbone of the FAdV-4 fiber1 knob is coloured magenta (**B**), while the EDSV fibre knob is coloured cyan. **E** The AA′ loop structures of the three FAdVs and the AB loops of the four CAR-binding HAdVs were compared. The AB loops of HAdV-2 (PDB code 1QHV), 5 (PDB code 1KNB), 12 (PDB code 1KAC) and HAdV37 (PDB code 1J12) are all coloured light grey. The AA′ loops of FAdV4 fiber1, CELO fiber1 and EDSV fibre are coloured magenta, yellow and green, respectively.

To further elucidate the receptor-binding property of the FAdV-4 fiber1 knob, its crystal structure was determined (Table 1). The crystal was obtained in 20% (w/v) PEG 4000 and 0.15 M potassium sulfate. The macromolecular structures generated during this study have been deposited in the PDB under accession code 7X5T. The structure was solved by molecular replacement using a monomer of the CELO long fibre knob structure as a search model. The asymmetric unit contains one chain, which can form a trimer by generating a symmetrical mate (Figure 2A). The knob domain of each monomer formed a compact β-sandwich with a typical topology similar to that of other known structures of adenovirus fibre knob domains. The β-sandwich is comprised of seven β-strands, ABCJ and HID (Figure 2B), the nomenclature of which was proposed by Xia et al. [13]. Moreover, when superimposed, the FAdV-4 fiber1 knob and the CELO fiber1 knob exhibited root mean square deviations (RMSDs) of only 1.469 Å (Figure 2C), and the FAdV-4 fiber1 knob and the EDSV fibre knob monomers had RMSD values of 1.316 Å (Figure 2D), which suggested that the three fibre knobs share almost identical structural folds. Taken together, these findings revealed that the three FAdV fibre knobs exhibited high sequence identity and similar structural conformations.

**Table 1:** **Crystallographic data and refinement statistics**

Crystal	FAdV-4 fiber1 knob
Data collection	
Space group	P432
Cell dimensions (length Å; angle °)	a = b = c = 116.6; α = γ = β = 90.0
Resolution (Å)	47.60–1.65 (1.68–1.65)^b^
R_merge_ (%)^a^	12.7 (32.95)^b^
Unique reflections	33,194 (1650)^b^
Completeness (%)	100.0 (99.2)^b^
<I/σ(I)>	27.1 (2.2)^b^
Redundancy	75.7 (79.0)^b^
Refinement	
Resolution (Å)	47.60–1.65 (1.68–1.65)^b^
R_work_/ R_free_ (%)	13.51/18.97
RMSD bond length (Å)/angle (°)	0.006/1.41
No. of molecules per asymmetric unit	1
No. of atoms	
Protein	1693
Water	215
Average B factors (Å^2^)	30.58
Ramachandran plot, residues in (%)	
Most favoured regions	98.0
Additional allowed regions	1.5
Disallowed regions	0.5
PDB code	7X5T

^a^R_merge_ = Σ(|I − <I>|)/Σ(I), where I is the observed intensity and <I> is the average intensity of all the measured observations equivalent to reflection I .

^b^Numbers in parentheses represent values in the highest resolution shell.

To further evaluate the CAR-binding properties of the three FAdVs, the structures were superimposed onto those of the human adenovirus type 2 (HAdV2) [14], HAdV5 [13], HAdV12 [15] and HAdV37 [16], which all bind CAR (Figure 2E). In these HAdVs, the most important section in terms of CAR binding is the AB loop. Studies have shown that these adenoviral loops may not be fully flexible variable regions but rather organized receptor engagement motifs with carefully evolved structures [17]. Alignment of these loops revealed different topologies in these critical receptor-interacting regions. The AB loops of FAdV-4 are most homologous to those of EDSV and CELO in terms of amino acid sequence identity and spatial alignment, respectively. Figure 2E shows the HAdV AB loops (in grey) and the AA′ loops of the three FAdVs. Indeed, the A strand of the three FAdV fibre knobs is longer than that of HAdVs when the extra A′ strand is present. As shown in Figure 2E, the AA′ loop is squeezed between the β strands and adopts a totally different conformation from that of the CAR-binding AB loops of these HAdVs. Therefore, the fibres of these three FAdVs should bind to CAR in a different manner than those of HAdVs.

### Analysis of the binding capacity of the FAdV-4 and CELO fibre knobs

**Figure 3:**
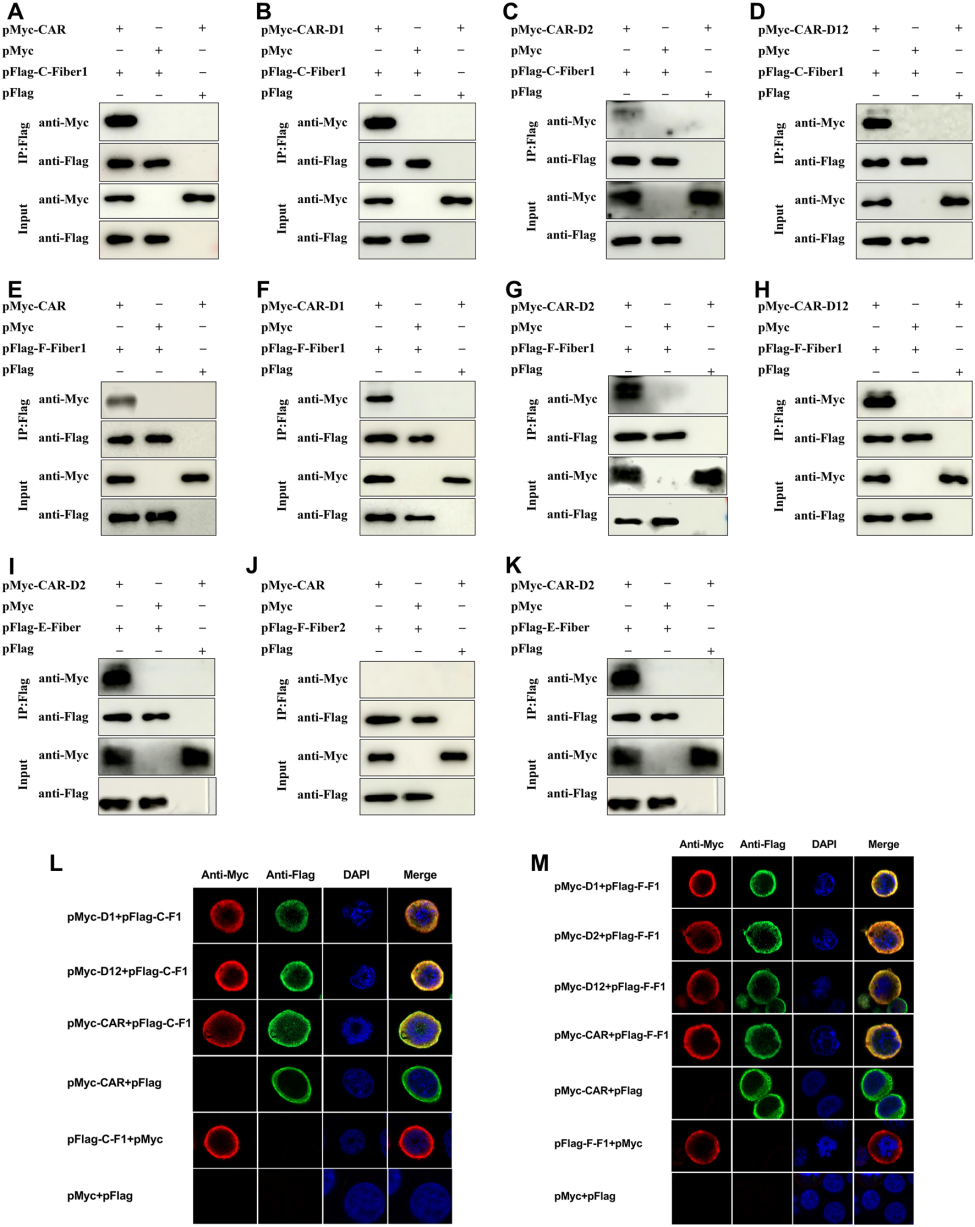
**Coimmunoprecipitation (co-IP) and colocalization analyses of CAR and the fibre knob.** **A**‒**K** Cells were co-transfected with pFlag-Fibre and pMyc-CAR using mouse anti-Flag and rabbit anti-Myc mAbs. After 36 h of incubation at 37 °C, immunoblotting analysis was performed to evaluate the binding of knob to different domains of CAR in the immunoprecipitants. **L**, **M** Analysis of the cell surface distribution and colocalization of CAR and fibre knob was performed by indirect immunofluorescence assay in CHO cells. The fibre protein (red fluorescence) colocalized with the CAR protein (green fluorescence) in the cytoplasm of 293T cells. The nuclei were labelled with DAPI (blue points).

To demonstrate the binding capacity of the FAdV-4 and CELO fibre knobs with CAR-D1, D2 and D12, we analysed the interaction using coimmunoprecipitation (co-IP) and confocal methods (Figure 3). Co-IP and confocal microscopy were performed as described by Song et al. [3]. The fibre head bound specifically to chicken CAR. These results confirm the findings of previous studies showing that the extracellular domain D12 is the main functional region of CARs. Surprisingly, both the D1 and D2 domains can be inhibited by the FAdV-4 fiber1 and EDSV fibre knob domains. However, the D2 domain was relatively less affected by the CELO fiber2 domain (Figures 3A–K). The FAdV-4 fiber1 knob protein (green fluorescence) was colocalized with the CAR D1 and D2 domains (red fluorescence) in the cytoplasm of CHO cells, which was different from the binding characteristics of CELO fiber1. Therefore, we concluded that the knob domains of the three representative avian adenoviruses can interact with the D1 domain of CAR, and the knob domains of EDSV fibre and FAdV-4 fiber1 can interact with the D2 domain of CAR. FAdV-4 fiber1 can bind to CAR, while fiber2 cannot (Figures 3L and M), confirming that fibre-1, but not fibre-2, is the key factor that directly mediates FAdV-4 infection [5].

### Binding affinity of the fibre knob to CAR

**Figure 4:**
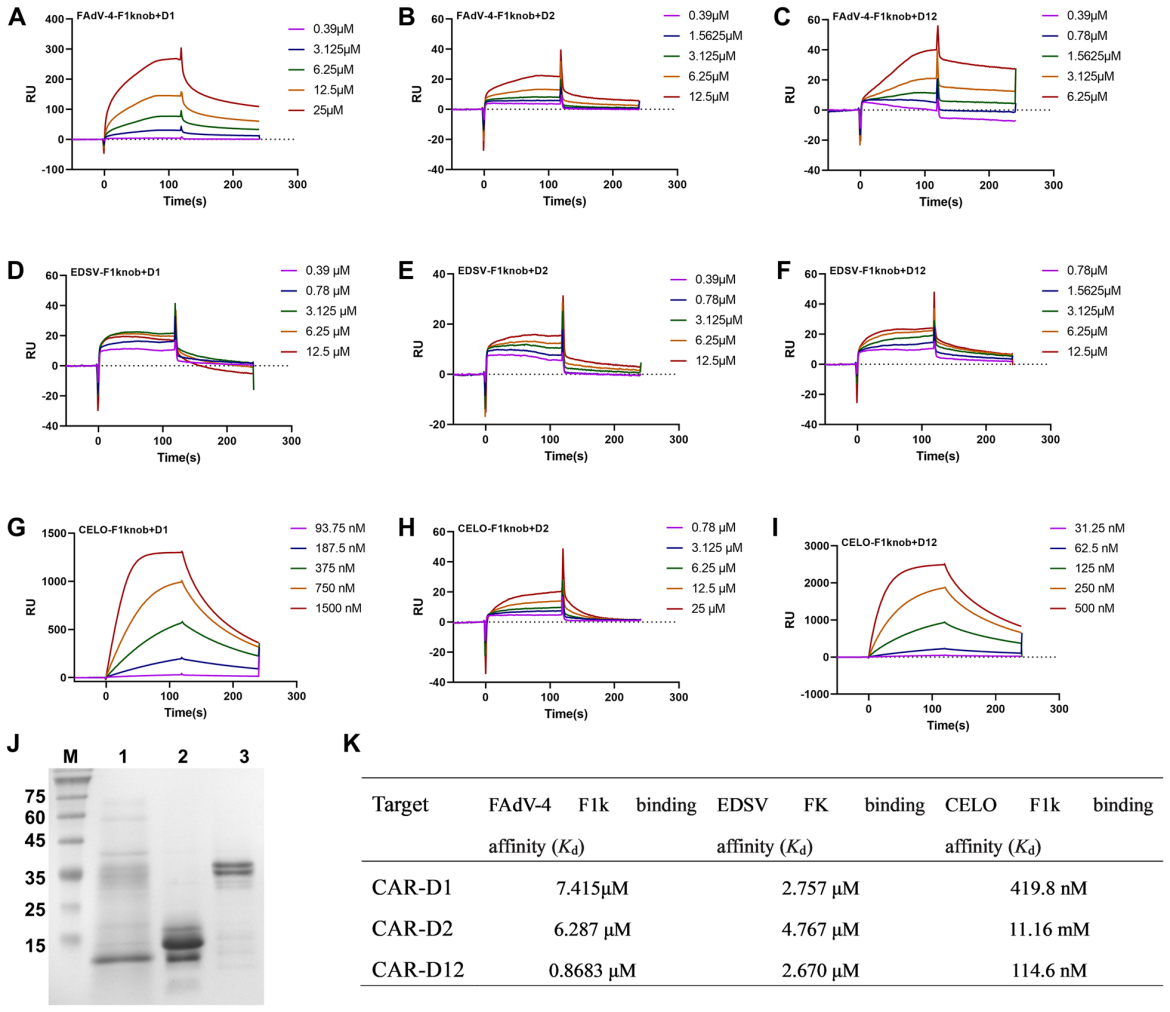
**The binding affinity between soluble CAR domains and fibre knobs was characterized by surface plasmon resonance (SPR).** The experimental curves (red lines) were fit using a 1:1 binding model (black lines). The equilibrium dissociation constant (Kd) was calculated by Biacore X100 Evaluation software. The results were calculated from the sensorgrams. The S.E. did not exceed 15% of the Kd.

To further investigate the direct interactions and determine the affinity between the various domains of CAR and the fibre knob of the three representative avian adenoviruses, surface plasmon resonance (SPR) analysis was performed using a Biacore X100 instrument system with a CM5 sensor chip. The soluble CAR D1 domain, D2 domain and D12 were expressed and purified (Figure 4). Figure 4 shows sensor grams of the interactions between fibre knobs with the CAR D1 domain, D2 domain and D12 domain. The binding affinities (Kd) of the purified FAdV-4 fiber1 knob protein for the D1 domain (7.415 µM), D2 domain (6.287 µM) and D12 domain (0.8683 µM) were comparable to those of the EDSV fibre knob (2.757 µM, 4.767 µM and 2.670 µM, respectively). Compared to those of the FAdV-4 fiber1 knob (Kd = 6.287 µM) and the EDSV fibre knob (Kd = 4.767 µM), the binding affinity of D2 to CELO fiber1 knobs (Kd = 11.16 mM) was 1000-fold weaker than that to the D1 domain (Figure 4K). Combined with the extremely fast kinetics, this suggests a highly unstable interface. The difference in the binding affinity of CAR to CELO and FAdV-4 fiber1 knob is likely due to the key residues on the surface of the fibre proteins. CAR-D12 (in solution) interacted with all three fibre knobs (immobilized) with low micromolar Kd and higher affinity, thus demonstrating the ability of the extracellular domain of CAR to directly bind to fibre knobs.

## Discussion

Most recent work has shown that representative strains of avian adenovirus tend to utilize CAR as a receptor and seem to exhibit diverse binding affinities and binding modes [4, 18]. Given the uncertainty regarding the receptor binding mode of CAR-T cells, this study was performed to examine the functional use of CAR-T cells in combination with three representative FAdVs. We solved the crystal structure of the FAdV-4 fiber1 knob at 1.6 Å resolution and found, as expected, that it is structurally similar to that of the long fibre knob of CELO (fiber1) and the EDSV fibre knob. The interaction between the fibre knob and different domains of CAR was verified by confocal and coimmunoprecipitation analyses.

Most of the available knowledge about adenovirus receptors is still based on the heavily investigated human adenovirus [19, 20]. The binding region and affinity of FADVs for CAR-T cells are significantly different from those of HAdVs. With a few exceptions, FAdVs are equipped with a single type of capsid fibre protein that interacts with receptors via its knob domain. The three representative FAdV (CELO, FAdV-4 and EDSV) fibre knobs exhibited high sequence identity and similar structural conformations. Although all three representative adenovirus fibre knobs maintain the same topology, differences in the length and orientation of the strands and loops may lead to significant functional differences in the external surfaces of the trimer unit.

Previous studies revealed that the adenovirus-binding activity of CAR is mainly localized in the D1 domain, but a lack of the D2 domain would reduce virus binding and infection. Recent findings in FAdVs highlight the fact that CAR is likely involved in viral invasion [3–5]. In this study, we compared representative avian adenoviruses to determine whether a similar binding mechanism with CAR was possible. All three avian adenovirus fibre proteins reacted with the CAR and CAR-D1 structural domains but differed in their binding to CAR-D2. We further confirmed that both the D1 and D2 domains of CAR contribute to the interactions with the fibre proteins EDSV and FAdV-4, while CELO mainly relies on the D1 domain. These results obtained by SPR are consistent with the results of co-IP and confocal analysis. However, these findings seem to be inconsistent with previous studies, which suggest that CAR-D2 is the active domain responsible for binding to the short fibre (fiber1) of the novel FAdV-4 [2]. Therefore, in terms of the interaction between the knobs and the CAR extracellular domain, FAV-4 is different from CELO and more similar to EDSV. Moreover, the results suggest that the CAR-binding affinity to the avian adenovirus fibre knob is much lower than that to human adenovirus fibre knobs [15].

Together with existing structural information, we described the similarities and differences among the three avian adenovirus fibre knobs in terms of their CAR-binding properties, revealing a much more sophisticated binding mechanism than that of HAdVs. However, further studies are required to analyse the critical residues involved in the interaction between FAdVs and CAR when FAdVs enter cells and determine whether an unknown receptor exists.

## Data Availability

The GenBank accession codes AGT17758.1 and AY597011 and the PDB accession codes 1J12, 1KAC, 1KNB, 1QHV, and 2IUN were used in this study. All the other data are available from the corresponding authors upon reasonable request.
